# Obstetrics and Obstetrical Surgery

**Published:** 1899-10

**Authors:** Eric Vondergoltz

**Affiliations:** New York


### HOMŒOPATHIC PHYSICIAN,

A MONTHLY JOURNAL OF

HOMŒOPATHIC MATERIA MEDICA AND CLINICAL MEDICINE.

"If our school ever gives up the strict inductive method of Hahnemann, we are lost, and deserve only to be mentioned as a caricature in the history of medicine."—CONSTANTINE HERING.

Vol. XIX. OCTOBER, 1899. No. 10.

### OBSTETRICS AND OBSTETRICAL SURGERY.

(Concluded from Sept. No., page 406.)

ERIC VONDERGOLTZ, M. D., NEW YORK.

If we are are going now over the different operations and their statistics we will find that the forceps were applied 165 times, as follows: Breech, 35; Head, 95; After-coming head, 35.

Once before I stated, in the *Medical Century*, No. 12, 1895, my position regarding the application of the head-forceps to the breech of the infant under due consideration of a logically-built forceps. Here I will shortly refer to the use of the forceps on the after-coming head, either after version or after the trunk and limbs are born in a case of breech-position. To confirm my position on this question, I will cite the *American Text-Book on Obstetrics*, p. 484: "But as cases frequently occur in which the head can be delivered with far greater ease by a rapid alternation between two or more methods than by the continued use of any one alone, it is for this reason, if for no other, well to be familiar with all the methods which have been found of value."

I am still satisfied of the correctness of my statement, although I remember that an obstetrician claimed to find it absolutely false when, in 1888, I was recommending the forceps on the after-coming head.

Shall all other methods be tried and used, until—the much-abused baby will be dead! In all these methods we see the manual traction play an important part—and this traction injures the spinal cord, etc., and so causes death to a larger proportion than the generally supposed suffocation. The absolutely condemning position of the late Carl Schroeder, of Berlin, echoed by H. Fehling in 1889, must be opposed, and is opposed by the authors of the *American Text-Book*, who are instinctively recommending the axis-traction forceps (without giving reasons). "Moreover, there are but few cases in which a skilled operator, aided by efficient suprapubic pressure, fails to deliver by manual extraction; but as some cases do occasionally occur, the forceps should always be at hand before the delivery is attempted. If forceps be used the body should be raised to a nearly vertical position, and the forceps should be passed into place upon the sides of the head, beneath the abdomen of the child."

The statement, "to have the forceps on hand," is a concession that the once-started operation must be abandoned and superseded by another one—on account of failure. This failure means loss of time, and so, unnecessary danger to the baby's life. Now every physician knows that every change in an operation is generally regarded dangerous and as proof of bad and hasty judgment.

I most emphatically believe that we must choose without hesitation such means in delivering a woman as will warrant surety of work, no danger to the mother, and the least danger and injury whatever to the baby.

My rule is the following: As soon as the trunk, etc., of the baby is delivered, and if the head is not following easily and immediately, no time is lost and the forceps is applied to finish the act of delivery. As I had to operate under the most unfavorable circumstances—being consulted after many trials and long delays—the results will be compared favorably to the statistics of H. Fehling (Vol. III, *Obstetrics*, Ed. P. Müller, 1889).

Case No. 450.—May 14th, 1896, Mrs. M., æt. 19, was in labor ten hours, with slow progress. Her attending physician sent for me at 1 A. M. As soon as I arrived at her home Dr. B. chloroformed the patient. Having prepared everything in the described way, patient was put on the transverse bed. The examination showed the breech firmly engaged in the superior strait of the pelvis. I applied the forceps without any difficulty. Having now delivered the body and disengaged the arms, I immediately applied the forceps to the head under the body, as the occiput occupied the left quadrant of the maternal pelvis. The baby was so easily delivered and rapidly, that Dr. B. and myself could observe the suffocative motions of the baby while bringing down the arms. The placenta was then easily brought down, and the perineal laceration was closed with four deep and six superficial silkworm sutures. Patient was discharged the fourteenth day.

Breech presentation in first part. on account of greatly contracted pelvis.

Case 640.—June 10th, 1896, Mrs. D., æt. 28, first part.; attended by a midwife. Patient was in labor for the last seventeen hours as I was called in. On examining, I found breech-presentation, back of the child occupying the posterior right pelvic quadrant. I immediately, after having made every preparation, quickly applied the forceps and delivered the baby (body inclusive of the arms, which were firmly retained). As that had cost much time, I applied the forceps to the after-coming head according to the rule to apply the forceps from the abdomen. With a difficult and steady traction I also delivered in this case a living child. The patient was under Chloroform during the operation, as also afterwards for the repair of the complete perineal tear. Patient was in my office for removal of the superficial silkworm sutures July 7th.

Under the same chapter regarding the forceps, I must refer to my way of doing episiotomy contrary to the teaching of most of the text-books. It is done in the median line, after the French mode (Chantreuil). The difference consists in this point, that I make the median incision while the forceps are applied and the outer part (the raphe) and the lowest part of fossa navicularis with the frenulum laborium are forming (by stretching to the utmost) an edge. This edge is incised with short strokes of a pair of scissors. Every further manipulation of the forceps will renew the appearance of the described edge, which then will be immediately relieved by one or two strokes with a pair of scissors. It must be remembered that the cutting will be done so even that, finally, the whole episiotomy will appear as done at once. So I was able to save many sphincters ani from rupture. The necessary closing of this artificial tear is not different from the operation of laceration. The reunion, perhaps, goes quicker as the surfaces will be coapted better than in general lacerations; so that lately I smooth the lacerated parts in perineorrhaphy before closing tears.

The repairing operation after delivery was done: trachelorrhaphy, 83; perineorrhaphy, 180; vaginal tears, 52; total, 315.

The immediate repair of the cervical tear was done by me in 1890, and published in *Med. Monatschrift*, Vol. II, No. 8, N. Y.

The operation will be generally easy, as the uterus in the most cases will be pushed so far down by pressure on the abdomen, that the lacerated cervix in front of the vulva will be repaired by one or two silkworm sutures. If the artificial descrusus uteri should be incomplete, the operation, with the aid of a speculum (Edebohl's) will be executed like any other secondary trachelorrhaphy.

I must remark regarding trachelorrhaphy after labor, that we have in this operation one of the only reliable remedies for hemorrhage post-partum.

The way to operate primary perineorrhaphy is a two-fold one, to proceed in the old way by different rows of interrupted sutures, or a running one, as described in the *N. Y. Hom. Jour. of Obs.*, 1895, No. 5.

Case No. 315 will illustrate a case of episiotomy, trachelorrhaphy, and perineorrhaphy after labor.—February 5th, 1895. Mrs. D., æt. 42, second parturition; first child born seventeen years ago—premature still-birth.

Pains began March 16th, 1895, about 10 A. M. and worked on slowly until March 17th, 2 A. M. Cervix then open for one finger; later, broke at 4 A. M.; pains stopped at 6 A. M. As patient was exhausted and cervix so far dilated that I could introduce cautiously the forceps, I began to operate. If during my traction the perineum stretched and formed the described edge, I slowly incised. By thus doing the sphincter ani, at least, was saved, but the laceration had extended so far into the vagina that finally the perineorrhaphy and repair of the vagina *portrier* required twenty-two sutures. The placenta came complete in twenty-five minutes by itself. In this case the uterus could not be brought down, so the repair of the cervix was done in the described way with the aid of the speculum.

The child (17 lbs.) was born nearly suffocated. Puerperium was uneventful; but patient discharged in perfect health, union post-partum, March 20, 1895.

To have together all operations which more or less are preparatory or afterwards needed, where the delivery of the infant was managed with instruments, I must make some remarks here about incisions in the rigid cervix—to gain room on the one side, but mostly to overcome the rigididty on the other side. If these incisions (bilateral), as I am accustomed, are not carried deeper than one-eighth to three-eighths of an inch, no sudden unmanageable tear will result. I prefer to cut with a somewhat curved blunt-pointed bistoury—the tears are securely closed afterwards with silkworm sutures. A possible cutting of a branch of the descendent arteria uterina will be of little concern. And the closure afterwards will be of less difficulty than if an irregularly-shaped tear would necessitate the primary trachelorrhaphy.

The following history explains well the foregoing remarks:

Case No. 381.—Mrs. B., æt. 23, third part.; in labor-pains for fourteen hours; cervix in all that time not more dilated than to be passed by the end of the fourth finger. As I was called in by Dr. G., I prepared patient in the most rigorous way, as I saw that the surroundings were of the most unfit condition; the room back of the small store was without light and ventilation. Patient was chloroformed and washed with five per cent. Lysol solution, and as I anticipated a considerably complicated laceration, I immediately shaved the vulva. After a second Lysol ablution and a most scrupulous vaginal syringing and rubbing, I performed, as described, the hysterostomatomy. The effect of this procedure is sometimes a miraculous one, and in three minutes the cervix was dilated so far that I could apply the forceps. The baby (male), of about ten pounds, was dead. The head was in the third position. The extraction was the most difficult ever met with, as the pelvis was narrowed by an osseus growth in the last five years.

Patient was under Chloroform from 9.30 A. M. to 12.20 P. M. The terrific post-partum hemorrhage was effectively checked after the artificial cervical tear and the complete perineal laceration was fully repaired. All in all, forty silkworm sutures were used.

For safety's sake a Lysol 0.5 per cent. gauze tampon was left in utero and vagina for the next twenty-four hours.

The delivery and all operative manipulations were done in a dark bed-room, with the attendance of husband and mother-in-law; the husband had to light my work with a not brightly shining oil-lamp.

Patient was under my care from September 16th to October 6th, 1895. The superficial cervix, vaginal and perineal sutures were removed October 1st.

Cephalotripry and emboly were performed seven times in all—without one single death, where generally the mortality is very high.

I cannot otherwise explain these good results than that in all cases I proceeded with the utmost care and most absolute antisepsis, combined with the fact that I closed, after completed operation, every minimal laceration of cervix and vagina, besides this (while the patient was under Chloroform) I made the most energetic use of the uterine curette with following hot intrauterine Lysol irrigation.

The most remarkable cure is the following, No. 155.—In the evening of January 10th, 1890, Dr. V. sent word for me to take charge of the confinement of a patient, as he was sick in bed.

Mrs. M. F., æt. 39; one child (girl) 4 years old. My examination revealed the following state: Cervix well dilated, head of the child in second (Bush) position, occiput in the right anterior pelvic quadrant. From the following morning at 9 A. M. pains became weak, and suddenly the heart sounds of the baby could not be auscultated any more.

As the patient grew weaker every hour, she consented to have another physician sent for to give Chloroform. She was readily brought under the anæsthetic, and after a careful examination I decided to try the version. I succeeded in bringing down the feet and so to have effected the version, until suddenly the abnormally large body of the infant was firmly inwedged. As the last resort, I eviscerated the dead infant and removed the contents of the thorax. By this time I had finally succeeded in bringing down the extraction to the arms. The bringing down of the upper extremities was tedious, but in some time was successful. The head having resisted all and every manœuvre, was finally perforated between neck and chin, the head having been raised, as the occiput was caught by the maternal symphysis pelvis. The perforation was comparatively easy. After having washed away some brain-matter I applied a strong Nægele forceps and delivered finally the patient. A complete laceration had resulted, going upward in the rectum two and a half inches.

Having detached the placenta, curetted the uterine cavity, and washed out most thoroughly, I immediately repaired the perineal laceration. This repair was done without Chloroform, as patient was extremely weak. All in all, twenty-five silkworm sutures were used. Patient made a good recovery; sutures were removed January 23d.

The narcosis was conducted by Dr. P. The operation lasted from 11 A. M. to 2.30 P. M. As the baby appeared to have been of an immense size all parts were put together on the scale, and with the loss of blood and brain, etc., the remnants of the body weighed 20.8 pounds.

Patient was in my office February 17th, 1890, and I took the following measurements: Conjugata area, 10.75 cms.; tr. (inlet), 9.0 cms.; tr. (outlet), 7.3 cms.; ant. part. outlet, 7.4 cms. From these data I would like to diagnose this pelvis as a narrow, funnel-shaped pelvis.

To-day I would perform, in this case, symphysiotomy.

Only a few words must be added in regard to the treatment of the 65 cases of abortus with two deaths—both in consultation. Since the publication (*Med. Monatschrift*, Bd. I, No. 6, 1889) I have changed so far in my treatment that I believe it rational to empty speedily the uterus of its contents without delay, especially if we remember that most patients, without many exceptions, have waited for some time. We must not forget that the external genitals and vagina are not in such a condition that we can safely leave the proceeding to itself. The *modus operandi* is the following:

Patient (with or without narcosis, corresponding to her nervousness and state of dilation of the cervix) is put in lithotomy position on a Kelly-pad on a table. Vulva and vagina are washed, and, if necessary, the vulva is shaved. A Simon or Edebohl's speculum is inserted, the cervix is steadied by a heavy (Skene) traculum-forceps, and, if, necessary, dilated to such a degree that the largest number of the required curettes will pass easily and freely. The best dilator, in my eyes, is Dylie's. I begin with an abundant intrauterine injection and then I begin to extract with the largest curette as many particles of the ovum as possible; as soon as the large débris are removed, I begin to use the next smaller curette to remove as thoroughly as possible all velamina. Finally, the smallest size of my curettes must remove as much as possible out of the tubor ostia.

This curettage is several times interrupted by an intrauterine injection. Finally, the vagina is loosely packed with 0.5 per cent. Lysol gauze for the next twenty-four hours.

Case 440.—April 16th, 1896, Mrs. S. A., æt. 18; first part., third month. Patient was treated for three days by a druggist of the neighborhood. As I saw her for the first time and heard this statement—besides that she was somewhat feverish (101.5 degrees; pulse, 120)—I sent immediately to Dr. B. to give Chloroform. As soon as patient was under the anæsthetic she was placed on a Kelly-pad in lithotomy position with the help of Dr. Edebohl's leg-holders.

Having cleaned (that means thoroughly washed) the external genitals and scrupulously syringed the vagina, and, after removal of all dried blood-coagula, I examined and found that the uterus was perfectly closed (on account of Ergot); the fetus was expelled, the placenta retained, recognized here by the torn and prolapsed umbilical cord. I then dilated carefully the cervix and curetted after a copious preliminary intrauterine irrigation. Finally, after having curetted as much as possible all débris, and as the irrigation fluid was returned absolutely pure, the vagina was packed loosely with Lysol gauze and the patient was restored to her bed. The next day temperature was 99 degrees and pulse 100. Patient was discharged the fifth day, cured.

Regarding symphysiotomy I will state that all my eight cases recovered, and that the reunion of the dissected bones was per perineum. I briefly describe the operation, as I intend to publish an essay on symphysiotomy later on.

*Instruments.*—I use a common scalpel, a slightly curved, narrow-bladed, blunt-pointed bistoury, Hagedorn needle-holder and curved needles, silkworm sutures, six hæmostatic forceps (déan), female silver catheter, and one-half per cent. Lysol gauze, adhesive rubber plaster, muslin binder.

*Assistants.*—Three assistants are necessary—but as in most cases I had only one, a brother-physician for the narcosis, I accustomed myself to operate with two lay assistants, generally two women, to do just what they were asked.

*Preparatory Operation.*—As soon as the patient is fully chloroformed she is put on the table, and the abdomen, pubes, and vulva are as fully prepared as for any celiotomy. For a short time the patient is posted in the lithiotomy position to clean and disinfect the vagina. Shortly now, before the patient is ready for the first step of the operation, I examine the mobility of the sacro-iliac joints.

*Operation.*—The patient is now put straight on the table with flexed legs but drawn apart. After the depression on the upper margin of the symphysis is found by the searching finger, and after the catheter is introduced into the urethra (to hold the neck of the urethra back and sideward—mostly to the right), the long incision is invariably made, terminating at the root of the clitoris. The retropubic tissues now are separated by the introduced index, by gliding and tearing down and behind the symphysis, and hooked under the inferior ligament. The curved, blunt-pointed knife is now introduced under guidance of the inserted index to the lower ligament to sever the structures of the ligament until the bones are felt to have separated. During this whole time both halves of the pelvis must be supported by the assistants to prevent a sudden giving away and injury to the fascia of the sacro-iliac joints.

As soon as the symphysis has been cut the woman is delivered as required by the concrete circumstances.

After delivery the symphysis is brought together by pressure on the trochanters. The wound is closed by two rows of silkworm sutures and the pelvis is immobilized by adhesive straps of rubber plaster helped with the muslin binder.

The dressing consists of my Lysol gauze. The after-treatment does not differ from any other aseptic vaginal after-treatment, especially regarding care of vaginal secretion.

The patient must be kept in bed in the unchanged posture on the back with closed and outstretched limbs.

The pelvic bandage must be worn from six to eight weeks.

*Manual Operations.*—In all manual operations I invariably perform the extraction of the infant immediately after version. And, consecutive to the condition of the patient, I deliver the placenta or wait for its natural detachment for thirty minutes, as I used to do in every case of spontaneous labor with self-development of the baby.

In all cases of manual extraction of the placenta, I curette and wash out the uterine cavity.

If in any way this procedure should be thought too rigorous I believe that the result, no death in 290 cases, speaks favorably for my method. I include in these 290 cases the detachment of placenta adhæsiva, etc.

In overlooking my 565 obstetrical cases, I make the following conclusions:

(1) That in private practice the physician must rely more on antisepsis and antiseptic preparations of the most rigorous manner to protect the patient most thoroughly.

(2) That the operation always must be modified by circumstances, and that absolutely normal types of operations in obstetrics cannot exist—on account of surroundings, assistance, etc.

(3) That any obstetrical operation can only be judged by the final result for mother and baby. Any theoretical criticism will finally prove fully irrelevant, like the criticism of symphysiotomy by H. Fehling in 1889, as: "I am sorry that I cannot omit in this place to refer to an old and nearly rightly-forgotten obstetrical operation, as it is revived by some Italian obstetricians."

The histories of the seven deaths of these 565 patients are following:

(1) Mrs. G., æt. 28, fourth part.; attended by a midwife for thirty-two hours. Fetus in transverse position. Version easy without narcosis. Died two hours later. Sepsis. Case No. 31.

(2) Mrs. R., æt. 28, third part.; attended by a midwife. Rigid os, head-presentation in third position. Version and extraction of a dead child. Died second day after delivery. Was in labor for thirty-six hours. Case No. 160.

(3) Mrs. E., æt. 25, third pregnancy; abortus of twelve weeks. Patient was treated by different physicians. Dr. D. called me in consultation. Patient had temperature of 102 degrees and pulse 160. Uterus enlarged, and vagina several placentar residues. I proposed, as the last resort, curettage. Curettage was done under Chloroform at 9 P. M.; patient seemed to improve. Temperature 100 degrees and pulse 120 at 1 A. M. Collapse and exitus at 9 A. M. Case No. 443.

(4) Mrs. M., æt. 31; attended by Dr. K. I was sent for at 8 P. M. to apply the forceps. Complete perineal laceration with following primary perineorrhaphy. Patient was well until fifth day (August 12th, 1896), 5 P. M. Patient did not show any signs of sepsis, but was suddenly suffering from the fearful heat. Patient anxious, dry skin (perspiration had stopped suddenly at 4 P. M.). I was in permanent attendance until exitus at 8 A. M., August 13th. Sunstroke. Case No. 477.

The deaths in my own practice were as follows:

(5) Mrs. O., æt. 37; abortus second month; fifth pregnancy. 'Sent for me the fifth day after blood began to go. As patient was very feverish I immediately recommended curettage; by consent I curetted the same day. The septic process was gone too far, and patient died from septicæmia the third day after curettage. Case No. 120.

(6) Mrs. G., æt. 28, fifth part.; easy self-development. Detachment of placenta. Thrombus and embolus. Death the third day. Case No. 212.

(7) Mrs. P., æt. 34, was sterile for nine years; finally successfully treated in Berlin (G.). Shoulder-presentation, vesion and extraction in narcosis. Everything went all right until the sixth day. Sudden exitus. Vitium cordis. The post-mortem examination proved: Insufficienta abortæ, hypertrophia cordis sinistri as cause of death. Case No. 236.

### THIRD PART.

In the foregoing pages several points could not be mentioned, as, for instance, placenta prævia, modified Turndelenburg's position, bandaging the abdomen, arrest of milk-secretion, etc., which in reproduction of the different histories cannot be omitted, if the description of obstetrics in private homes *versus* hospital work shall be considered complete.

*Placenta prævia* was encountered fourteen times without one death. In all cases the version of the fetus with following extraction was done. These fourteen cases must be divided in two groups: Placenta prævia centralis, 9; Placenta prævia lateralis, 5 (left side of the mother, 2; right side of the mother, 3).

I encountered one case of placenta prævia lateralis sinistra in which undoubtedly I could observe that the lateral position caused a marked slowness of the labor-pains, resulting from a certain degree of uterine paralysis going out from the seat of the placenta (*Med. Monats.*, Vol. I, No. 11). Since that time I have carefully investigated the seat of the placenta in every slower-going labor, and to-day I believe firmly that the progress of the labor is directly proportional to the seat of the placenta—if nearer down to the internal os, slower and more irregular, the labor will be found equal to tendency of uterine hemorrhage intra-partum. It must be stated further that the seat of the placenta to the right or to the left of the uterus wall is nearly equally distributed and of no special consequence.

Case No. 137.—Monday, September 8th, 1889, I was called to a first parturition with generally contracted pelvis. The vaginal examination showed the external os cervices partly passable for three fingers. Head was in third (Bush) position. Water-bag was ruptured five hours ago. The pains growing stronger without any result. The carefully repeated examination revealed that the posterior lip of the cervix appeared paralyzed like a fold of thin skin dangling down.

The observation that the dilation did not proceed while the pains had more and more the character of tetanic contractions, and finally that the heart of the fetus began to become weaker caused me to execute the version with following extraction in Chloroform narcosis. During my dilation of the cervix and introduction of the hand into the uterus I found that immediately over the perplexing posterior cervical lip the placenta was literally inverted (from *Med. Monatsch.*, Vol. I, No. 2).

Case No. 476.—Mrs. K., æt. 46 years, fourteenth part., had different uterine hemorrages during pregnancy (treated by different physicians). Labor expected August 18th, 1896. I was called August 15th for a sudden and copious hemorrhage (without any pain). Uterus was closed. The vagina was filled with coagulated clots, which I removed. As patient was in a half-ugly, half-tearful humor, I gave Cham., expecting to see some dilation while waiting. At my next visit, three hours later, patient rested comfortably; during this whole time no pains.

At 11 P. M. I was called on account of a new hemorrhage. I found the patient nearly bled to death. Hurriedly I sent for Dr. S. in the neighborhood to give Chloroform. As soon as narcosis was complete, the patient was put on the transverse bed. The cervix was dilated manually. As I passed the internal os I found a cavity like a vestibule filled with coagulated blood; on the left side of the intrauterine wall the over-reaching margin of the placenta could be felt through the velamina. The whole ovum was intact. The fetus was in transverse position. I now ruptured the velamina, turned on the feet, and extracted the child. Immediately the placenta was removed and the uterine cavity carefully curetted. Patient was in a very low condition after the operation.

The puerperium was uneventful and I discharged the patient August 29th. Mother and baby (girl of eleven and one-half pounds) in good condition.

Trendelenburg's position, or a modification of it, has been used by me several times, and, as I must confess, without intention the first time. Only the casual observation caused me afterwards to use the elevation of the pelvis with full deliberation. The following history will illustrate fully the foregoing remark:

Case No. 117.—Mrs. Ch. B., æt. 30, first part. I was called at 2 A. M., March 21st, 1892. I found the fetus in breech-position, half-way, firmly engaged in the pelvic bones. My efforts to push the fetus upward, for bringing down the feet, was as unsuccessful as was that to bring down the baby by the hook manœuvre. As a last resort, I tried the forceps.

The bed-room was of such small dimensions that there was no room for other furniture besides the bed. If now the patient was on the transverse bed, there was absolutely no room in front of her for me to stand while applying the forceps.

As the bed had a low foot-end I placed a board incline over this foot-end with the patient in narcosis on it. I had expected from my foregoing examination severe difficulties in applying the instrument. My surprise was considerable on finding the breech relatively disengaged, so that I did not encounter any difficulty; nay, I may state that if it had not been for the sake of false criticism, I am positive that in this position the bringing down of the feet would have been an easy task. Since that time I have had several occasions where the elevation of the pelvis on account of the rules of gravitation worked as a great help, especially in versions.

Case No. 172.—Mrs. R., æt. 22, first part.; attended by a midwife. I found during examination face-presentation in the transverse diameter. As the midwife had administered some Ergot and the face was considerably deep engraved, I arranged the Trendelenburg's position and could now easily effect the version.

*Bandaging of the Abdomen.*—As a rule, I am against bandaging, as the muscles will be paralyzed and the lymphatic stream in removing the deposit of accumulated fat during pregnancy will be prevented. It is my custom to bandage only the very pendulous and large abdomen of very stout women, more for the personal comfort of the patients than for any other reason. The actual harm of a bandage will be illustrated in the following history:

Case No. 138 (Vide *Med. Monatsch.*, Vol. I, No. 11).—Called by a midwife the fourth day after delivery, I found the patient with red, flushed face, laboring breath, brilliantly shining eyes, and tortured by singultus without cessation, headaches, vertigo and fear.

The physical examination proved a temperature of 103

degrees, pulse much accelerated, vagina hot and dry, no lochiæ, uterus sensitive, hard and contracted, os cervicis firmly closed. As patient complained much about the binder I allowed her to have it removed.

Two hours later the lochiæ began, and the next day all symptoms had disappeared, and the temperature was 98.2 degrees.

It was clearly the retention of the lochiæ effected by the binder, especially the personal observation of the patient, who stated that soon after the removal she felt beginning relief, and before the prescribed medicine had reached her, the temperature with dyspnœa, etc., began to go down; besides, there are several cases on my records of similar observation; and, finally, nobody believes that such a puerperal fever (on first sight) could be cured so quickly.

*Arrest of milk-production* in cases of still-birth, etc., besides the medical treatment was successfully established by enforced diet. That means that for the first five to eight days the woman must be put on smallest rations to urge the constitution to use up all material which under other conditions would be transformed in milk. This regimen, starting on the first moment, prevents all after treatment.

*Narcosis*, as best material in my cases proved Chloroform. That to this day Chloroform is not regarded as highly as deserved can be understood from the fact of generally wrong administration. To the present day I cannot see the evil results of Chloroform after the second state, as Lusk mentions. It may be stated here that I firmly believe that such an opinion results from the bad administration by unskilled assistants; as Ether is easier given.

LITERATURE.

Lusk, Science and Art of Midwifery, 1889.

Müller, Handbuch d. Gebartshülfe, 1889, Vol. III.

Schrœder, Lehrbuch d. Gebartshülfe, 1886.

System of Obstetrics, by Hirst, 2 Vol., 1888.

Text-book of Obstetrics, by Morris, 1895.
Vondergoltz, Ether or Chloroform, Med. Prene., Vol. V, No. 3.
Vondergoltz, Obstetrical Notes, Med. Prene., Vol. VI, No. 6.
Vondergoltz, Med. Monatsch., Vol. I, No. 6.
Vondergoltz, Med. Monatsch., Vol. I, No. 2.
Vondergoltz, Med. Monatsch., Vol. II, No. 8.
Vondergoltz, Med. Monatsch., Vol. II, No. 10.
Vondergoltz, Med. Monatsch., Vol. III, No. 9.
Vondergoltz, Med. Monatsch., Vol. III, No. 10.
New Method of Primary Perineorrhaphy, Hom. Journal of Obstetrics, 1895, No. 5.
Forceps in Breech-position, Med. Century, 1895, No. 12.
The Clinical Value of the Creosol Preparations, N. Y. Med Times, 1896. No. 12.

*New York City.*

